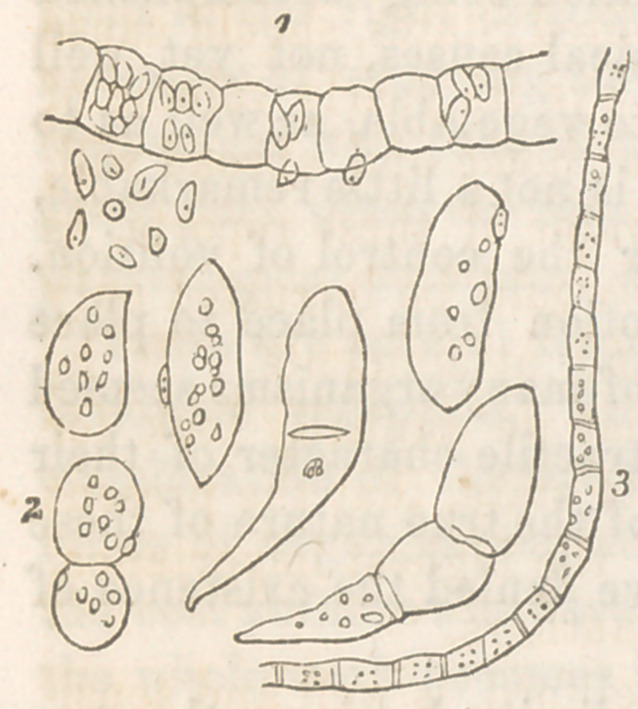# Bibliographical Notices

**Published:** 1854-05

**Authors:** 


### BIBLIOGRAPHICAL NOTICES.

*Pneumonia: Its supposed connection, Pathological and Etiological, with Autumnal Fevers; including an Inquiry into the Existence and Morbid Agency of Malaria.* By R. La Roche, M. D., &c., &c.

(Continued from page 234.)

The work of Dr. La Roche was suggested, or rather he began to write it, in consequence of the revival of an opinion that pneumonia and autumnal fevers are connected by community of cause, viz., malaria. He sets about combating this heresy, and does it with entire success. The wonder will be, in the minds of some, that a grave refutation should have been necessary. He shows that these diseases prevail under different circumstances, as regards climate, seasons, winds and localities. Their occasionally coincident occurrence, and, at times, their successive appearance, have led some into the common fallacy of assuming affinity in the first case, and the relation of cause and effect in the second. It is even conceivable, and in fact the thing has presented itself, viz., that certain localities may undergo such changes of atmospheric influence at particular seasons as to alter their climate; so that the air and temperature of low grounds in winter may give rise to pneumonia, and the air and temperature of high lands in the latter part of summer and autumn, combining with some unknown epidemical influence, may cause fevers; but these changes or conversions do not militate against the position taken in the present work. There is no mutation of causes, but merely a difference, for the time being, in the seat of their production.

The author has somewhat too readily enlarged the list of those writers who advocate the community of cause, or malarious origin, both of fevers and pneumonia. Dr. Bell certainly was not of the number, when he says: "Why, then, demand miasm as a cause of fever and refuse it as a cause of pulmonary disorders?" He meant to make the argument a *reductio ad absurdum;* and that as nobody, he thought, could attribute these disorders to a miasm, or any single peculiar morbid agency, so, neither ought fevers to be supposed to have any such specific or exclusive origin; for if they have their distinctive characters, so have, equally, pulmonary disorders. A long list of writers of eminence is given by Dr. La Roche, beginning with Morton and ending with Bailly, "who describe a pneumonic form of intermittent fever, or periodic form of pulmonary inflammation, produced by the same cause as ordinary intermittents, and thereby, like the preceding writers, acknowledge the identity of both sets of phenomena represented in the compound." A perusal of Bailly's work left the impression on our minds, that inflammation of the lungs, liver, or spleen was separate in its nature from the fever which affected the system at the same time—an addition and complication which destroy the patient, although the fever, as far, at least, as regards its periodicity, had been removed by the administration of quinine or other anti-periodic remedies. This writer entertains, we think, the same opinion as that so emphatically expressed by the author himself, in the latter part of the volume, (Chap. VII.,) in which he speaks of cases of pneumonia marked by symptoms appertaining to autumnal fevers, as the results of complications. He denies that we are justified in admitting the identity of the two diseases represented in the compound phenomena, which he had just before noticed, or that the one is a modification, or really and substantially nothing more than a peculiar form of the other. All these cases he believes to furnish "illustrations of the complications or co-existence of two distinct complaints, produced by distinct causes, having distinct seats and characters, and being governed by different laws, but which often modify each other to a greater or less extent."

We cannot follow the author in his citation and analysis of those parts of the works of the different writers which bear on the subject, but must refer our readers to the volume itself, for a perusal of the whole line of argument, which they will find to be conclusive on the point at issue, and which sheds much light on collateral or incidental topics—a praise which, with some qualification, is merited throughout the entire volume itself. Many interesting notices of the medical topography of different places, both in our own and foreign countries, are introduced and seasonably applied.

The frequent reference to malaria as a cause of autumnal and periodical fevers in general, led the author to an inquiry into the reality of such a cause, on which some have thrown doubts, and from a belief in which others have formally dissented. This is the theme of the second, third and fourth chapters of the volume; and it is discussed with a fulness of detail and extent of research unequalled in any other work. The contents of these chapters are well calculated to shake the faith, or rather remove the doubts of the non-conformists, who, by the way, are sometimes rebuked in rather a trenchant manner. It is not, however, always easy for even the most practised fencer, when he has touched his adversary again and again with the point of his rapier on a vital part, to avoid exclaiming "a hit, a palpable hit;" and even insinuating that the passage at arms was foolishly sought by the defeated party. He passes in review all the different states in which marshes are found—as flooded, merely moist, in process of desiccation and quite dry; and the modifications imprinted, by these several states, on the incumbent and contiguous air, contrasting, in these respects, town with country, and showing the effects of draining. He inquires, also, into the alleged changes in the atmospheric air over marshes, caused either by the diminution or the increase of some of its elements, or the addition of others, as far as pneumatic and analytical chemistry and the microscope can supply us with any evidence in the matter.

The different explanations of the etiology of periodical fevers by those who do not hold to the doctrine of malaria, are successively examined and analyzed, and dismissed as inadequate.

The author (in Chap. V.) resumes the main argument, by comparing pneumonia and autumnal fever, in reference to their causes, mode of progression, symptoms, anatomical characters, and the circumstances by which they are influenced; and, after due inquiry and comparison, he shows that they differ from each other in all these particulars. In the next and concluding chapter, he points out the fact that, although pneumonia and autumnal fevers are independent of each other, as regards their nature and causes, they may still combine together, and form, like other complaints, hybrid diseases, which must not be considered as peculiar forms of fever. These several propositions are enforced and illustrated with the copiousness of pertinent facts, derived from a great variety of sources, which distinguish the other portions of the volume.

Having now exhibited the boundaries and chief divisions of the field surveyed by the author, let us follow him in his description of some, at least, of its characteristic features, especially those relating to malaria or miasm, and indulge at the same in a few passing remarks on the light in which he views them. He believes that Hippocrates recognized the existence of miasm as a separate morbific agent; but the language of the Coan sage does not justify this inference, although he describes clearly the nature of the localities and the impure water, which, he tells us, give origin to periodical fevers, and which, in the modern creed, are believed to generate miasm. Of the general fact, that the contemporaries and successors of Hippocrates in Greece were well aware of the deleterious nature of the air from marshes, and that they sometimes spoke their belief in allegory, sometimes in plainer description, we cannot doubt. We even go farther, and admit that they did not attempt to explain the occurrence of the diseases incidental to such localities as those now adverted to, by supposing them to be the products of changes in the sensible qualities of the atmosphere alone; but still we cannot show without very strained construction of their language, that they either asserted or understood the existence of a separate and specific cause such as miasm, or malaria. Hence, we think the author has gone in advance of his evidence, by assuming that which is not proved, when, in a subsequent chapter, page 290, he speaks of Hippocrates telling us that malaria predisposes to ulcers; we shall quote the entire sentence. "In many southern regions, the West India Islands, for example, malaria predisposes, as Hippocrates informs us was the case in Greece, to ulcers." Again, at p. 329, he says, in the first of a series of propositions growing out of a survey of all the facts and statements which he passed in review: "1. The doctrine of malaria, though of ancient origin, and very generally admitted, has encountered and continues to encounter opposition." But this is merely a literary question, and, whichever way construed, does not affect the main argument, for the support of which the author has abundant evidence in modern writers, without doing any violence of construction to the language of the ancient ones. Among the Roman writers cited are, Lucretius, Virgil, Varro, Columella, Celsus and Livy; and the Greeks, in the Roman period, Diodorus Siculus, Strabo, Dionysius, Dion Cassius and Paulus Ægineta.

In modern times Fernelius must have the credit "for having been the first, after the revival of letters, to show the influence of contaminated air in the production of epidemics, and, as M. Rochoux has well remarked, (p. 125,) laid down the doctrine of miasmatic infection in a way which leaves scarcely any thing to desire." A number of other writers are named in succession by the author, including Prosper Alpinus, who practised in Egypt from 1580 to 1584, and who ascribed the plague of that country to morbid exhalations. Ramazzini, at Modena, and Bontius, at Batavia, were equally explicit in pointing out marshy places from which arose putrid exhalations. Sylvius (de la Boe,) at Leyden, Hoffman, Baglivi, Chirac and Porzio are mentioned in this connection. "Finally, Lancisi, collecting all the facts and information already possessed, and adding many he himself had amassed during a period of observation of thirty years, made them illustrate the etiology of epidemic and endemic diseases in general, which he ascribes, as every one knows, to marsh and other exhalations."

The author, after saying that the agency of malaria is not universally admitted, betakes himself to the task of enumerating the dissidents from his creed, and the objections made by them. In reply, he tells us, that the appearance of fever where there are no marshes does not disprove the agency of malaria, since other sources of supply and emanation of malaria are admitted by the most intelligent writers, Lancisi among the number, who yet believe in its being the specific cause of periodical fevers, intermittent and remittent, with their varieties and complications. This admission, however, of plurality and diversity of source, we might say manufacture, complicates the question which the advocates of malaria are required to solve.

"*The non-detection of malaria in the atmosphere no proof of its non-existence and agency.*"—Under this proposition the author admits as true, in the main, the fact, that, not only has the existence of malaria or specially noxious effluvia not been proved, but, still more, that the air of marshes contains the same proportion of constituents which enter into the composition of the air of the highest mountains, and, also, that no deleterious gases beyond some very limited, we might say accidental evolution, have been found in the air over marshes or other malarious localities. The observations on this head are given with considerable minuteness.

"*Fever not due to the action of any known gases.*"—This almost follows as a corollary from the preceding proposition, and is admitted to its fullest extent by the author; but he contests the assertion that nothing is found in the atmosphere of sickly localities. He shows that Moscati, on submitting the water condensed from that which had been dissolved in the atmosphere of insalubrious places, such as of that over rice grounds and in the wards of the large hospital of Milan, found deposits of a flocculent matter emitting a cadaverous odor; and Brocchi, at Rome, detected albuminous flakes (animal matter) in the dews of the Pontine marshes. Similar results were obtained by Vauquelin, on analyzing the dews from the marshes of Languedoc and Provence, while *fresh* dews gave a somewhat, though it must be confessed not an essentially, different product, viz., alkaline salts with vegetable and animal substances. "Julia de Fontenelle and Herpin obtained results differing but little from those recorded by Brocchi, so far as regards the flocculent or inorganic matter, while in common dew nothing of the kind was discovered." The air of marshes, as we are told by the first of these writers, if kept six months, acquires a nauseous smell, an effect not noticed in common air. Dumas, and before him Volta, found an organic substance combined with the gases disengaged from stagnant water. Ozanam and Boussingault's experiments are to the same effect; and Thenard and Dupuytren discovered that "the carburetted hydrogen obtained from marshy grounds, when passed through water, deposited therein a peculiar putrescible matter; a result not obtained from the same gas disengaged in the ordinary way." M. Gasparin states, that on his condensing fog and dews, "a peculiar matter was obtained which, on trial, was found detrimental to health and fatal to sheep. The same results were obtained by Mr. Malagutti, an Italian chemist." Dr. Hume, of Charleston, S. C., discovered an organic matter suspended in the atmosphere of localities infected with the poison of the yellow fever. It was supposed by the experimenter to be a mixture of animal and vegetable matter. Facts of this nature are worth recording, but they have not been of that uniform occurrence, nor always so indicative of associated disease, as to induce a belief in the substances above noticed, or "azotised flakes in the atmosphere" being the cause of malarial fevers, while, at the same time, their presence may not unreasonably be supposed to have some hand in the production of these diseases.

The force of the objection to a belief in the existence of malaria, based on the inability of chemists to detect it in the atmosphere, is obviated, the author believes, and quite plausibly too, by their want of success in discovering other morbid products— the matter of the contagion of small pox, for instance, in the atmosphere. "In addition, it may be remarked, that medicines, even purgatives, are sometimes found to act through the medium of the air, in which they cannot, on analysis, be detected."

"*Nature and condition of fever localities lead to the opinion of the existence of malaria,*" is a proposition laid down by the author which he illustrates with his customary copiousness of literary reference and details of medical topography. His perspicuity is, we think, however, at fault on this occasion, in his assuming as proved the very point at issue. The question is not whether certain localities are the chief homes of fever. Nobody disputes this; but whether these places are sickly by the generation in them of a particular febrile poison, is the question to be solved. Goitre is endemial in certain districts of country; but although there is still, among inquirers into its etiology, a difference of opinion as to the means by which it is produced, some attributing it to the use of waters of a particular quality by the inhabitants, others to the air which they breathe and the food which they eat, few, we were going to say none,* have attempted to cut the knot by referring this disease, marked as it is by such distinctive traits, to a single principle, and calling it a peculiar poison. The thing may be so; but very certainly it is not proved by our specifying a list of localities, of an analogous character, in which goitre prevails.

In the accuracy of the following paragraph we place full reliance, as the expression of what we believe to be an important truth;

* For want of a better explanation, a causeof this nature has been supposed, by alate writer, to be operative in the production of goitre.

but beyond this we lack the means of pushing our etiological explanations. The author had been describing marshes and low grounds as the home of periodical fevers. He goes on to say:

"Nor is it less certain that the yellow fever is traced almost invariably to city districts, noted for filth and improper ventilation, in the vicinity of ships, docks or wharves, to narrow and confined courts and alleys not far from there, to collections of substances animal and vegetable, in a state of decomposition, &c. Instances to that effect have been too frequently observed, and are too well authenticated to be denied; while the connection between the existence of places of the particular kind mentioned, and the appearance of that form of fever, is too constantly found to prevail, to be considered simply in the light of a coincidence." The agency of sustained high temperature to give effect to this kind of locality in the production of yellow fever, is dwelt on by the author in another part of the volume.

"*The danger of an attack of fever increased in proportion to proximity to such localities*," is another proposition of great practical moment; and it is suitably enforced by the author, in a series of facts recorded by different writers. We were about to introduce, as we have repeatedly proposed to ourselves to do at different times heretofore, extracts from the pages of this volume, which teem with useful knowledge, on points under discussion; but we are as often restrained by the reflection that no quotation, or series of quotations, can do justice to the unbroken series of ample researches made by the author. He has given us not so much a mine to be worked as an overflowing treasury, from which every medical reader ought to hasten to enrich himself. We have not, ourselves, been indifferent students, nor have we passed our time without thinking and reading, and enjoying a fair share of experimental observation on the main subjects of this volume; and we would, therefore, ask some faith to be reposed in our opinion, when we say how we rejoice over its pages now, and how we would have fairly gloated on them in early life, when we were greedily seeking for literary food of this kind. When, therefore, we shall be found, at any time, in the remarks we have yet to make, merely enunciating the views of the author, it may be taken for granted that, in every instance, there is a large body of valuable facts, and often cogent conclusions, which we have not had time or room to introduce.

Passing over, for the present, the negative side of the argument, in which the author adduces a number of interesting facts adverse to an explanation of the origin of autumnal fevers from extremes and vicissitudes of the sensible states of the atmosphere, we shall follow him on the line of his evidence in chief. He adduces, in proof of the existence and morbid agency of malaria, "occurrences on shipboard," and, the strongest fact on his side, "the causes of fever wafted by winds passing over sickly localities." The references to occurrences of this nature, in parts of France, Italy, South America and of Asia and its islands, will be new to most of our readers; nor are those in our own country less interesting. Facts in large number and pertinent comments are introduced, respecting the effects on the health of the inhabitants in the districts respectively, in which drying, overflowing and reclaiming marshes, clearing and drainage, have been practised. Apropos to this branch of the subject is the fact, that "as cities enlarge and improve, malarial fevers decrease in them." The beneficial effects of draining marshes and covering sickly places with water, are pointed out as leading circumstances to the belief inculcated by the author.

The argument is continued in the next chapter (IV.) by a reference to the salutary "effect of the washing of sickly places," and their geological formation and the plants growing in them. Again, "the cause more effective near the surface of the earth than at a distance;" and it is "mitigated or destroyed by sanative measures," and "arrested by trees, &c." "Fever arrested by removing sources of infection," and it is "sometimes connected with the existence of certain fogs or mists." "The stagnant water of marshes injurious to health," whenever it is used as a drink, and its deleterious influence on animal life, is further shown in fish not being able to live in it. We omitted a proposition in an early part of this chapter, viz., that "some forms of malarial fevers resemble diseases produced by putrid substances introduced into the circulation." The "effect of a paludal atmosphere on the duration of life," also manifested by "an impress on the lower order of animals and vegetables," in common with the human being placed under its influence, is indicated and enlarged on, with the usual abundance of illustrative facts. Less stress can be laid on "the great diffusion and mortality of autumnal fevers, and the violence of their attacks, as evidence of "the cause being a gaseous poison." "The cause of autumnal fever extends its action to the fœtus *in utero*;" it "appears to be neutralized by the poison of some zymotic diseases which have no effect on common complaints," and is "antagonistic to that of some other diseases," as, for example, of typhoid fever. On this point the author enlarges in a very instructive, we were going to say edifying manner. It is one that interests all country practitioners, and ought of itself to prompt every one of them to a perusal of the volume.

We revert now to the negative which the author puts on the explanations of the etiology of periodic fever deduced from sensible states and mutations of the atmosphere. Notwithstanding all that has been said to the contrary by a number of writers quoted by him, he thinks "we may safely affirm that excessive, great, or long-continued heat will not do so," viz., account for the febrile phenomena; and a little further on (p. 136) he adds: "This want of necessary connection, as cause and effect, between high atmospherical heat and fever—common autumnal and yellow—has been pointed out by a large number of writers on these diseases, as they show themselves in various parts of the globe." The author, at the moment of writing this sentence, forgot what he had said in preceding pages, (68—70,) when speaking of pneumonia and autumnal fevers as prevailing at different seasons. He had just declared malarial fever to be, in our latitudes, especially, a disease of autumn; seldom appearing before the middle or close of summer, and ceasing on the accession of winter. "It is, emphatically, a disease of hot weather, requiring for its production a continuance, for some time previous, of high atmospheric heat. It appears, generally, some weeks after the hottest month, the period being retarded as we proceed north. For the same reason it may readily be understood to be a disease of hot latitudes, prevailing as it does violently and almost perpetually within the tropics, and ceasing long before we reach the polar circle." Speaking of the fever of Senegal, on the African coast, and the season in which the endemic is most rife, he tells us: "It is at this period (the hottest of the year) that remittent fevers annually make their appearance.* In the West Indies, the period of the greatest liability is between July and December, when the hottest weather, combined with considerable moisture, prevails." After references of a similar nature to the fevers of Bengal and Ceylon, he concludes in the following terms: "In a word, the epoch of appearance and disappearance may vary in different localities, according to the situation of those, and their position relative to the equator, and the consequent modification in the period of the seasons; but every where endemic or autumnal fevers break out, or are most rife during or shortly after the hottest weather." This must be regarded as tolerably emphatic testimony in proof of the relations, etiologically considered, between high heat as a cause, and periodical fever, in its most aggravated forms, as an effect.

In the very next paragraph to that just quoted we read: "So far as regards the yellow fever, the disease may still more appropriately be denominated one of hot weather, requiring, as it does, a higher average temperature during the summer months—not less than between 76 and 80 degrees (Fahr.) It manifests itself in no climate where the temperature is below that average, and ceases to appear before we reach the limits assigned to ordinary periodic fevers." Dr. Drake's opinion, repeated by the author in a preceding page, was, that a summer temperature of sixty degrees is necessary to the production of periodical fever. Taking the data, as laid down in the above extract and in the opinion just cited, can we shut our eyes to the very significant fact, that fever, from simple intermittent to the highly aggravated remittent, and the still more dreaded and malignant yellow fever, increases in violence and danger in almost a fixed ratio to the increment and duration of atmospheric heat. That this heat is only operative under certain conditions of locality is admitted by all parties. The difference of opinion among them consists in some, like the author of the volume before us, insisting on the evolution, from the locality of the disease, of a specific poison, or a miasm, the action of which on the animal economy predisposes it to be affected by high heat, which is therefore, in their eyes only, an exciting cause. Sometimes, again, this proposition is inverted, and high heat is declared to be the predisposing, and

* Levacher, 48.

exposure to the miasm the exciting cause of the fever. Others contend that we need not speculate about the existence of a morbid agent, miasm, which confessedly would be inoperative, and remain for an indefinite period dormant, without the agency of heat, which is in fact, the *causa sine qua non.* In this latter view of the question, high heat acting for some time on the system, induces such a state of the functions as to throw it open to the fever, after exposure to any deviation from the laws of health. Among these we may instance being chilled in the night air, a debauch at table, or other sensual excess, loss of sleep, mental anxiety and especially fear, bodily fatigue, &c.

The author continuing his propositions, tells us: "Fevers are not the effects of humidity alone," nor are their occurrence accounted for by a "high dew point;" and, also, heat and humidity combined are "not the efficient cause of fever." The same is said of vicissitudes of temperature. "The attack is sometimes too sudden, and follows on too transient an exposure to infected regions, without appreciable atmospheric vicissitudes, to be the effect of these or any other kindred cause." "Fever not the effect of particular electrical states of the atmosphere." The facts accumulated by the author under the preceding heads lead, he thinks, to the belief in a poisonous agent floating in the atmosphere. He does not believe that the innocuousness of some marshes, and of localities similar to those that are sickly, can be adduced as proof of the non-existence or non-agency of malaria. "The exemption may be explained in various ways," as by "elevation;" "degree of heat;" "sheltering from the action of the sun;" "free ventilation;" "humidity of the soil."

We have now stated, in the form of a series of propositions, with occasionally an enforcing fact, the heads of the argument contained in Dr. La Roche's rich volume, to show, first, the difference, in their etiological and pathological relations, between pneumonia and autumnal fevers, and, secondly, the existence and morbid agency of malaria in the production of the last mentioned class of diseases. In the first or main theme, we entirely concur in opinion with the author. The second or collateral, the theory of malaria, does not carry with it the same conviction to our minds as it probably will to the greater number of his readers. It is not our intention at this time to present any counter theory, involving a different explanation of the etiology of periodical fevers, nor to discuss the subject at length; and we shall close this article with a very brief allusion to some of the difficulties in the way of admission of a belief in malaria, or marsh miasm.

At the outset, we are required by the advocates of the malarial theory, and unquestionably by the author himself, to admit a variety of miasms: thus, when speaking of the diversity of effects produced, he says, "poisons extracted from various sources of infection differ materially in composition and nature." (p. 324.) The exhalations which produce yellow fever, he adds, never produce intermittent fever. "Those from swamps, marshes, &c., give rise to periodic fevers of different grades, never to yellow fever, properly so called." Allusion is made to typhoid relapsing and typhus fevers, as resulting from diversified exhalations of some sort. "Even among fevers that are, strictly speaking, of malarial origin, some difference occurs in their symptomological and other characters." Writers are named from Lancisi to Dr. J. M. Smith and Monfalcon, who, severally, refer to, approve, or insist upon a probable diversity in the nature and composition of the miasms in question, as exhibited by the diversity of the phenomena they produce.

Dr. La Roche, in common with many other writers, believes that the miasm or malaria causing autumnal fevers is a gaseous poison. Admitting the premises just now stated, and we have several gaseous poisons, one notably for yellow fever. But as far as any approach has been made by chemistry and the microscope, to detect a poison or poisons in the air of a sickly locality, it has been of a solid substance of a vegeto-animal nature, and not a gas; and this poisonous matter has not been met with to an extent or with a uniformity at all commensurate with the range of periodical fevers. We need hardly say that, as no known gas is believed to give rise, either by its deficiency or excess, to the fevers in question, so neither has any new gas been detected which can be ranked as a cause of the occurrence of these diseases.

We revert to the probable influence of abnormal states of the atmosphere—extremes and vicissitudes—in the production of fevers which are commonly regarded as of malarial or miasmatic origin. Their etiological value is denied by Dr. La Roche; but from his own abundant records, made to elicit truth and not to maintain a system, we find evidence of a strong character in favor of the affirmative view. Of the leading part performed by heat in giving rise to periodical fevers of the worst grade and to yellow fever, we have spoken in a preceding page. The author devotes a section to show the "bad effects of copious rains followed by great and desiccating heat," and among other illustrations relates what befel the French army in the Morea. "On their landing there in 1828, the French troops were exposed to intense heat by day and cold by night. These vicissitudes, succeeding to a long period of dryness, invited the approach of the annual epidemic. Rain fell in heavy showers on the 18th and 19th of September, and the disease broke out on the 20th."* After describing the extensive and varied surface of a locality in the Province of Rio Janeiro, (Brazil,) which suffered extensively from periodical fever in 1830, he closes by the remark: "The epidemic was evidently due to a great drought which occurred in 1829—30, and succeeded to heavy rains which flooded the country.

"It may be observed, in illustration of this," he continues, "that sickly seasons are generally those in which a wet spring is followed by a hot summer." p. 234—5.

We now direct attention to those states of the atmosphere manifested by winds, which, both by their duration and their alternation, exert a decided influence over the animal economy. Here, again, we draw on the prolific stores accumulated in the work before us. The author is speaking of the appearance of pneumonia and of autumnal fevers "under the influence of opposite winds." Dr. Bone is quoted to show how yellow fever "prevails in Brimstone Hill, St. Kitts, when the strong winds that have swept foul ground on Mount Misere impinge upon the persons in the ill-constructed barracks and out-buildings on that hill." But in the sentence immediately following, and which we produce, winds and rain are spoken of as direct morbid causes, without the qualifying circumstance of "foul ground;" "and in Tobago, Dominica, Grenada, St. Vincent, and on all the hilly,

* Gouraud-Etudes sur les Fievres Intermittentes pernicieuses dans les Contrèes Meridionales, p. 218.

uncleared islands of the West Indies, strong north and east winds, impinging upon the troops and their families in ill-constructed barracks, are causes of the disease." We ought to have adverted to some statements of the author on a preceding page to that just quoted from, (73.) He repeats, without discrediting the supposition, that Edinburgh obtains fever through the agency of the east wind which blows it from Holland. Although endowed with a tolerably robust faith, we confess ourselves unable to receive this transmarine evidence of the existence and diffusion of malaria. Whoever has suffered, as we have, from the infliction of an east wind when on the German ocean itself, while crossing in a good old fashioned slow sailing ship from England to Holland, in the month of April, will not seek for any additional aid in the way of causation of fever; other circumstances of predisposition from season, &c., favoring its operation. The east wind is eminently unfriendly to us here at home, on this side of the Atlantic, and will develop intermittent and other periodical fever as speedily as in Great Britain; but we do not attempt to explain its effects by making it the bearer of miasmata, either from the marshes of Holland, the fens of the east of England, or the bogs of Ireland. The fact is, that the east wind is inimical to health all the world round, whether it blows from land or water. "In Batavia, the north-east wind, which is very prevalent during July, August and September, is highly unfavorable to health." This wind must, however, owing to the geographical position of Batavia, on the north side of the island, blow from the sea.

More emphatic testimony to show the causative power of winds in producing yellow fever could scarcely be borne, than is contained in the following language, held by M. Lefort, and quoted in the present volume (p. 75,) with the remark that his ample experience and accuracy of observation cannot be doubted. "The development of the yellow fever, in the West Indies, in a great number of men at the same time, in different parts, at a distance from each other, on a level with the sea, or slightly above, on board vessels at port or at sea, coincides so exactly with the prevalence of the south winds, that it is impossible not to recognize in these meteorological conditions the true cause of the epidemic of yellow fever. The effect here is intimately and necessarily connected with its cause. The production and extension of yellow fever under the prolonged influence of the south winds, is a fact observed by every one ; so inevitable indeed, that it can be produced without fear of being ever mistaken. This action of the south wind is felt here by every thing that breathes. They produce undefinable effects on our senses. We feel them in bed, or sitting at the desk ; they enervate, cause oppression and depress the spirits. To say in what these atmospherical alterations, the effects of which are so much to be dreaded, consist, and to seek to determine their specific nature, is, doubtless, a task beyond the faculty of man." We are disposed to adopt the conclusion reached by Dr. Lefort, and to receive as an ultimate fact the recorded influence of these winds, without an attempt at farther explanation, which is apt to mystify, by our supposing them to be the bearer of miasmata.

While thus making use of some of the facts so industriously and faithfully collected by Dr. La Roche in the present volume, we must not omit to state, that he adduces them with a different signification and in a different connection from those which we give to them. According to his view, if we do not mistake him, all the atmospherical influences which operate so prejudicially on the human system, are only means or secondary causes for developing into downright disease, a morbid predisposition induced by the depression of malaria or marsh miasm, on which last alone depend, he thinks, the distinctive febrile phenomena. This miasm, supposed to be of vegeto-animal origin, and familiarly spoken of as a poison, is, however, unlike all known vegetable and animal poisons. They are the products of organic forces, of living organization, in fact, whereas, this is assumed to be the product of decaying and dead organic matter, so that all the analogies drawn from those fail of application to this. Many of them may be brought to a state of extreme division, and so volatilized and diffused through the atmosphere as to be no longer cognizable by our senses ; but still they are, each of them, reducible to matter in either a fluid or a solid form, and we know their first homes and origin. Miasm, on the other hand, always escapes our senses, is always volatile, or, if condensed, it is only so by inference, never by demonstration. Other poisons, including those of the inorganic class, as the mineral ones, produce their peculiar or specific effects on the human system, irrespective of any modifying agency or circumstance, although some of them, as in the instances of the poisons of small pox, measles and scarlatina, take effect with more readiness, and on a much larger number of subjects, in virtue of the operation of modifying, and chiefly atmospheric, or, as we commonly call them, epidemic influences. But, on the contrary, in the case of marsh poison, it is in a great measure, if not entirely, harmless, by the admission of the most intelligent of those who advocate its febrificant power, until the human system, in which it rests as in a nidus, during what is called a period of incubation, is acted on by atmospherical and other known and appreciable causes. The marsh poison would remain in the living body as inert and inactive as the seed of the Egyptian wheat which remained for three thousand years in a state of desiccation in a mummy, and would only germinate into the forms of fever, as the latter would into leaves and stem, and productive grain, under the fostering influences of atmospherical exposure and modifications; moderate and few, indeed, in the case of the grain, but more marked and various in that of the miasm; assuming as we now do, for the sake of the argument, the actuality of this latter.

If we have recourse to the test of prophylaxis, the influence of malaria would seem to be reduced almost to that of a homœopathic dilution. In the same so called malarial district, during the latter part of summer and autumn, how different the fate of those who are well clothed, well lodged, and protected from the hot sun by day and the cool and damp air by night, and who are not fatigued by labor or excess of bodily exercise, from that of the poorer class who are exposed during a day of labor to great heat, and, from improvidence or necessity, afterwards, to the coolness and dews of night, without their bodies being protected from either extreme by suitable, and especially, woollen garments next to their skin; the food of these people, all the time, being often defective in quantity and of a bad quality, and their drink impure water, or worse still, alcoholic liquors. Now, be it remembered, that both these classes inhale the same air, which is supposed to be now largely charged, if not saturated, with miasm, evolved after some fashion or another from the surface of the soil; but yet how contrasted the effects of this miasm on these two classes. The first, or wealthy, escape almost entirely; the latter, or the laboring, suffer from periodical fever in large numbers. This is not an imaginary picture; it is a description from real life, made by Folchi, of the inhabitants of the dreaded Campagna, in the vicinity of Rome. Some of our readers have known, we dare say, from their own observation, others from competent authority, instances of families living in the midst of a district deemed to be eminently exposed to malaria, in fact in the centre of marshes, the individuals of which enjoyed immunity from fever, by living in such a way as to conform to the rules of health, in which, under the circumstances of their locality, are included the preservation of an equable temperature, and avoidance of humidity, in the house, by means of a small fire morning and evening, even in the hot months. During all this time, they were obliged to inhale the miasmatic air with which they were, according to the popular creed, surrounded. Can we, therefore, explain their exemption from fever in any other way than by their avoiding those atmospherical extremes and vicissitudes on which we have seen it, by the acknowledgment of the miasmatists themselves, mainly to depend? We may ask, in addition, if there is any known poison, the product of nature or compounded by art, which can be made so harmless, comparatively, by adherence to the rules of hygiene?

It must be remembered, that all the conditions for the formation and development of miasm imply changes and vicissitudes in the known qualities of the atmosphere. Autumn, the season admitted to be that in which the alleged poison is most active, is the one, also, which, by its reduced temperature and greater humidity, contrasts strongly with the summer season that immediately preceded it. But in a still greater degree must the human body be affected by the sudden contrasts between the hot sun of an autumnal day and the coolness, amounting often to coldness, and the accompanying heavy dews of an autumnal night. We have here not only extremes, but alternation of extremes, the impression of which is much increased on those who had been thrown into a state of indirect debility and of irritation by the influence of the protracted and intense heat of the preceding summer, and who are, in consequence, predisposed to the operation of any subsequent noxious agency. In our endeavors to explain the etiology of periodical fevers, the whole atmospheric machinery should be considered, and not merely one or two of its parts. It will then be seen that none of the circumstances under which miasm is said to be generated and rendered more active in producing disease, are unaccompanied by atmospherical extremes or vicissitudes, or by both. A recurrence of the fever in the spring, or in damp or rainy weather in winter, is easily explicable by our admitting, what is actually the case, a renewal of those atmospherical conditions, but with less intensity, which caused the disease in the preceding autumn, without our imagining that a certain poison, called miasm, had been lurking all the time in the system, and is now brought into activity. If it be said that the etiological explanation, on atmospherical data, sometimes fails, the same may be said, with equal force, of the miasmatic doctrine; inasmuch as we sometimes see periodical fever in districts in which the conditions for the evolution of miasm are wanting, and, again, an absence of fever where this alleged poison might be supposed to abound. Certainly there are, every now and then, instances in which no proportion exists between the amount and diffusion of the assumed cause and the violence and complications of effect.

Ample confirmation of these averments is furnished in the volume before us (p. 178—184.) The explanations of these anomalies from the miasmatic rule may, Dr. La Roche believes, be found in the elevation of the place so affected above the level of the sea; or, in other cases, to the absence of a sufficiently high and long continued atmospheric heat; or, it may be, to a very perfect and constant ventilation and a very rarified and pure atmosphere; on occasions "it may be explained by the peculiar geological character of the soil; the quantity and the quality of the surface water; or the proportion of sulphates the latter holds in solution. Sometimes, also, it is due to the rapidity of the river currents, the excessive and rapid dryness of the atmosphere during the hot season, the existence and extensive prevalence of refreshing and purifying winds, and often to the degree of desiccation of the surface by natural or artificial means, the degree of cultivation to which it has been carried, and other agencies of like import, as well as by the extent to which it is sheltered by rich foliage, and other means, from the action of the sun." After this enumeration of the various circumstances, most of them consisting in evident and avowed modifications of the atmosphere, and equally evident though less frequent phenomena depending on earth and water, which render null, and void of injurious operation, the so much dreaded subtle poison of miasm, there would seem to be no great stretch of skepticism in doubting the existence of any such deleterious agency. Its introduction in the causation of fever might be regarded as a speculative refinement, analogous to the sentimental view of the operation of love in certain cases; as in that of a man who was represented to have died, in one of the West India islands, of love—and yellow fever; and of another, in our own State, whose life was brought to a premature close by disappointed love—and hourly imbibing raw whiskey, as it flowed from the still.

In giving the main share to the atmosphere in the production of periodical fever, we would not be understood to overlook the operation of other causes, such as improper ingesta, including bad water, and perturbations, especially depressed states, of the nervous system. There are many cases on record of intermittent fever being brought on by indigestible substances; and some are related by Giannini, and others of the like effect being produced by irritation of the urethra, from introducing a bougie in cases of stricture. These are not regarded by Dr. La Roche as examples of true fever, although the paroxysms certainly simulated the febrile ones very closely, and recurred at similar intervals; and like these latter require the administration of bark, or of quinine for their removal.

Although not intended to produce that effect, the mention of some of the difficulties in the way of a solution of the problem of the etiology of periodical and yellow fevers, will, we dare say, cause a more lively desire on the part of our readers to possess the richly stored volume which has elicited the above remarks, and to peruse its pages in the same conscientious and pains-taking spirit in which it was written. They will then acknowledge that, even in the warmth of regard for the author personally, we have not indulged in any strained eulogy of the merits of his present performance; and they will, we are sure, join us most heartily in the wish that he would complete at an early day that "labor of years," of which he speaks in his letter to Dr. Meigs. The profession would then, every where, at home and abroad, be able, with a feeling of just pride, to point to the most learned, comprehensive and accurate work on Yellow Fever which has ever appeared. In such labors, varied by the indulgence of his refined literary tastes, the author can hardly fail to find rich consolation under bodily infirmities, which prevented him from early occupying a larger field of practice than is opened to most young men. Even as it is, he turns his few acres to better clinical acaccount than many who may be found boasting of the extent of their medical domain.

---

*A Treatise on Venereal Disease.* By A. VIDAL (DE CASSIS), Surgeon of the Venereal Hospital of Paris; author of the Traité de Pathologie Externe et de Médicine Opératoire, etc., etc. With Colored Plates. *Translated and Edited by* GEO. C. BLACKMAN, M. D., Fellow of the Royal Medical and Chirurgical Society of London, etc., etc. New York. S. & W. Wood. 499 pp. 8vo.

It cannot be denied that the doctrines advocated, if not originated by Ricord, have been constantly growing in favor. Whether original with himself or properly belonging to others, it must be admitted that he has done more than any other man to establish the truth of what he teaches. No one can undertake to make himself master of the subject of Venereal Disease without finding that he has to wade through much that is tedious, unsatisfactory and contradictory, and when he opens Ricord and reads his positive annunciations, his clear and abundant proofs, and his direct conclusions, the chaos of conflicting opinions disappears, and he feels that he has emerged from darkness into light, from theories and dogmas to facts and laws.

The influence of Ricord's teachings has been felt by all, who are not wedded to preconceived views, or who have not the candor to investigate truths which claim to be fully demonstrated by experiment.

It seems almost cruel to throw a doubt upon opinions which are now becoming a part of the definite and positive in medicine; it is like taking a step backward to unsettle those principles which we were just receiving as axioms, to intimate that Ricord is an authority no longer decisive as to doctrines or modes of practice; to learn that "the immutable *laws of experiment*," proclaimed by M. Ricord, "were annulled by experiment itself," and "the promised certainty resulted in uncertainty." Yet when such an authority as Vidal, the author of Traité de Pathologie Externe, the surgeon at l'Hôpital du Midi and the Lourcine, the successor of Cullerier, comes before the public, in a modest and dignified style, and offers, with true philosophic spirit, to discuss questions to which it was impossible for him to be indifferent, on account of the vast theatre of observation and experience in which he had so long been industriously engaged, we are bound to pause before adopting as final any preconceived opinions.

This work comes to us with no flourish of trumpets, no claims to discoveries of new laws, but with the honest endeavor "to collect the practical truths which are the results of observation, and those which have survived the downfall of systems;" he enters upon his work with no promises of leading men to conclusions with unerring uncertainty, but, after testing experiment by experiment, he says, "still I have thought it my duty to warn the young practitioner against disappointments and regrets, there being nothing, in my opinion, more dangerous, either in study or in practice, than to invest mere forms, and to pronounce that to be infallible which can be but probability or uncertainty."

We come now to the examination of the doctrines held by the author upon some of the mooted points with which the subject abounds.

*Identity of Gonorrhœal and Syphilitic Matter.*—Does he believe with Hunter that there is but one virus which produces blenorrhagia or chancre, according as it comes in contact with mucous membrane or skin; or with B. Bell and Hernandez, that there is one virus for blenorrhagia and another for chancre; or with Ricord, that blenorrhagia is a mere urethritis, a non-virulent affection, which can be produced at pleasure by irritants, and that there is but one poison, which always produces a chancre and never a blenorrhagia, and that the cases of chancres which have resulted from blenorrhagic pus, are to be all clearly explained by the supposition of there being a concealed chancre? Upon this vexed question, M. Vidal takes the ground that gonorrhœa and chancre are the results of the action of the same virus, and that both are followed by secondary affections; he states that there will always exist a disease of the mucous membranes, without ulceration, occurring under the same circumstances as chancre, caused by contagion, which is a specific disease, and not a simple phlegmasia, and which gives rise not only to primitive effects, but also produces in the same individual metastatic affections, such as opthalmic and arthritic inflammations;* and he quotes Messrs. Cazenave, Martius, and Legendre as asserting that "blenorrhagia produces as many cutaneous affections as chancre." He does not believe in the concealed chancre of Ricord. "I myself do not admit the existence of chancre beyond the navicula fossa." "I am aware of what has been written concerning the deep-seated urethral chancre; some pathological specimens of which have appeared satisfactory to those who exhibited them.

"But I must confess, after a very careful examination of that regarded the most important in proving the existence of a profound urethral chancre, I am convinced that this is a case not of syphilitic but of tubercular ulceration, which existed at the same time, in the form of cavities in the prostate gland, in the testicle, and in other organs of the same patient. I observe that some excellent surgeons partake of the same doubts." He then quotes Velpeau in his communication to the Academy of Medicine, Oct. 12, 1852, who thus remarks: "The specimens presented by M. Ricord, as examples of chancre, are far from being incontestible. In one of them I recognise that of a tubercular young man, having large cavities in the prostate portion of the urethra, and I see no indication that chancre had existed there. The other is that of an old man almost equally obscure. I saw these specimens when first exhibited to the Academy, but they were far from satisfying me of the existence of chancre."

He does not consider the discharges produced by mechanical irritation, or chemical solutions, as true gonorrhœas, because they are not followed by the secondary effects consequent upon the applications of "virulent pus, which is the medium of the syphilitic virus, and is, in my opinion, the most frequent and powerful cause of blenorrhagia." "We come then, to the conclusion of Benjamin Bell, and admit a doubt

* Pp. 21—22, Introduction.

virus, or more properly speaking, we apply two names to the same virus."

Although our author gives us experiments of his own to prove that chancre and its sequelæ result from the application of the gonorrhœal virus, still he quotes M. Baumes, who states that in five cases of simple blenorrhagia, in which he was certain there was no chancre in the urethra, constitutional symptoms followed, such as well marked, rounded ulcers in the tonsils; mucous tubercle at the commissures of the lips, about the anus, and in the scrotum; syphilitic ecthyma; furfuraceous, squamous, and papular eruptions.

M. Vidal goes still further, and states that syphilitic virus is the most common cause of *balanitis*, although he admits it may be produced by various other causes, such as want of cleanliness. He believes, too, that balanitis may be followed by secondaries, and that a balanitic discharge may be successfully inoculated. "Experience has shown that balanitis in both its forms, that is, without and with erosions, has been followed by consecutive accidents, which revealed the existence of constitutional infection. Experiments have corroborated these remarks; they have established the fact, that the matter secreted in a mucous balanoposthitis may be successfully inoculated, and that, too, when there is no ulceration, nor the slightest erosion, or solution of continuity in the mucous membrane." "These facts, however, have occurred at the *Hôpital du Midi;* they have been collected by an *interne* with whom I am acquainted, and have been inserted in his inaugural thesis. Dr. Bartholi asserts even that he has met with thirty cases of this kind. One of these is very remarkable; in a case of balanitis, without the slightest exfoliation of the mucous membrane, without any other venereal symptom, an adenitis supervened, which was treated by numerous punctures. Each puncture became transformed into a chancre. Pus was taken from one of these ulcerations, and, inoculated, produced what is called at the *Hôpital du Midi* the *pustule characteristique*, in other words, that which precedes chancre. (The *Thesis* of M. Bartholi was defended at Paris, in 1845; it was on the subject of *Syphilis* and Scrofula. I have not repeated these experiments, because the syphilitic nature of balanitis, under the form which is called simple *catarrhal*, I have never doubted, since I have been able to watch for a length of time the patients in whom it occurs."—p. 169.

We think that the above extracts show sufficiently the views of the author upon this important question, but his opening paragraph to the chapter on Chancre is still more decided.

"The syphilitic virus, when brought into contact, under certain conditions, with the living tissues, produces two principal effects: first, a form of inflammation, which is called blennorrhagia, from its product; second, a form of ulceration, which is *chancre.* I have already traced the history of blenorrhagia; chancre will form the subject of our present investigations. It is one of importance, and worthy of the reader's serious attention, particularly as certain syphilògraphers regard *chancre* as the only medium by which syphilis can be admitted into the system, as the first condition and the sine quâ non of the pox. Hence the doctrine, that *without a chancre there can be no pox.* Facts and the most legitimate analogy show the error of this proposition."—p. 190.

*Incubation.*—Upon another point of great interest, both in a doctrinal and practical point of view, M. Vidal holds opinions entirely at variance with Ricord. The question in dispute is, whether the virus acts locally, and then, after a certain time, is absorbed at once, and the local phenomena are only an evidence of the constitutional affection.

Ricord believes that a chancre has a local incubation, and his opponents believe in a general incubation. He considers that when pus is applied to a part, it requires several days to mature a chancre, and that it may be cut short in its progress by cauterization; and constitutional disease be thus prevented. Vidal sees, with Bosquet, great analogy between the vaccine and syphilitic virus. They believe that the infection is absorbed at once, and that a chancre is as much an evidence of constitutional affection, as a vaccine pustule is an evidence of the system having been affected by vaccination. Ricord answers this vaccine analogy, and states, that the cow-pock and tartar emetic pustule are analogous, and that emesis ought to be expected before the latter pustule is produced with as much certainty as protection from small pox-could be, before the pustule is matured.

Vidal says,—"I believe in incubation, that is to say, I believe that the virus may remain some time a cause without effect, at least, any appreciable effect." "The advocates of incubation appeal to analogy and to carefully observed facts. Thus the greater number of morbid poisons remain in the system for a certain length of time, without any evident external manifestation; they are there in a state of incubation. Why may not the syphilitic virus obey the same law? But facts favor this opinion still more than analogy." He then goes on to give cases in his own experience and that of others corroborative of this opinion.

*Transmissibility of Secondary or Consecutive Disease.*—Here our author and Ricord are directly opposed. Vidal not only believes in the communicability of secondaries from one individual to another, not hereditarily, but also is very positive as to the direct contagiousness of mucous tubercle, and that it is almost universally acknowledged that it may be a primary accident, and that Ricord is almost the only writer on syphilis who denies its contagious nature. And in support of these views he gives not only experiments but cases.

He believes that nurses with secondary syphilis may, through their milk, be the sources of disease in children. In all such cases Ricord thinks that such manifestations in the children may be referred, either to primary disease in the nurse, and the matter having been brought in contact with the child, which first maturates a chancre and then has secondaries, or that its constitutional taint may be accounted for by hereditary causes.

As to the syphilitic diathesis, which Ricord believes to be established by an indurated chancre not cauterized, Vidal does not concur in it. He believes that a person may have a true chancre and become perfectly sound; that he may have a secondary eruption and entirely recover; that he again may have a chancre and another eruption, and still recover.

*Syphilization.*—Two of the most recent defenders of syphilization have two objects in view—prophylaxis and cure. They not only believe that saturating the system with syphilis by inoculating chancrous pus renders the system insusceptible to primary and secondary accidents, as vaccination guards against small pox, but also that the virus is the best remedy against the action of the virus. With reference to this subject, which has excited so much interest recently on the continent, through the experiment performed on young M. Laval, a German physician, who believed himself to be completely saturated and challenged inoculation, he with great propriety remarks that "it would be irrational and inhuman to give positively to an individual a disease to protect him from that to which he is not fatally condemned. Prophylactic syphilization, therefore, is an irrational project." "As to curative syphilization, I have carefully examined the arguments, pro et contra, and in my estimation they are yet insufficient to decide the question."

We should like to bring before our readers still further the opinions of the author, and to place them in contrast with those of his competitors at the Hospital du Midi, but the limits of this notice prevent any further portrayal of his views. We should take much pleasure in exhibiting his modes of treatment, but must content ourselves with simply informing the reader that he is a merculiarist.

The work is published in a very handsome style, and beautifully illustrated by colored lithographs and wood-cuts. It is one of the most valuable works that has recently been issued from the press. Not only to the advocates of particular doctrines, but to every reader who wishes to be well informed upon this wide-spread calamity, will this work be a valuable accession.

The Editor, Dr. Blackman, has performed his duties with credit. The translation is generally good, and his notes are spirited, and add much interest to the text.

---

*Comparative Anatomy.* By C. H. SIEBOLD and H. STANNIUS. *Translated from the German, and edited with Notes and Additions*, by WALDO J. BURNETT, M. D. Boston: Gould & Lincoln.

The first volume of this work on the "Anatomy of the Invertebrata," has just been published; and after a careful examination of its contents, we most earnestly recommend it to the attention of naturalists, and to those members of our profession, who take an interest in such matters. The whole work is a translation of the "Lehrbuch der vergleichenden anatomie" of Von Siebold and Stannius, a treatise already well and favorably known to anatomists throughout Europe.

To those engaged in microscopical researches, this volume will be truly welcome. Within the last few years, our knowledge of the structure of living beings has been greatly advanced by the use of the microscope. Anatomy and physiology have been established on the more philosophical basis of histology, and contributions to histological science are sure of a favorable reception. On account of the small size of most of the Invertebrata, the microscope has been freely used, and the details of what is invisible in their structure, have been carefully recorded. In his preface, Von Siebold says, that it is only by such aid "we are able to show that this or that organ is a branchia, a liver, a kidney, an ovary or a testicle."

We know that there are many among us, who are devoted to microscopical investigations, and we cannot offer a stronger recommendation of this volume on the "Anatomy of the Invertibrata," than to say it abounds with an immense mass of new and beautiful researches into the anatomy and functions of those myriad organisms, which the miscroscope reveals.

The science of comparative anatomy is too much neglected by the members of our profession. It is intimately associated with all correct knowledge of the human organism. Man may be truly regarded as a microcosm of that life which is peculiar to our planet. Every organ and function which in him attain its highest degree of developement, exist in one or other of the humbler tribes of animated beings beneath him. This idea, first propounded by John Hunter, has been abundantly confirmed by the researches of later physiologists. They have successfully established the law, that the progressive phrases of embryo life correspond to the abiding forms, which are preserved in the total organism of animated nature, as typical of its gradative evolution; and that as the embryo of each higher animal, passes rapidly through the forms of the animals inferior to it, in order to attain its maturity and specific rank of being, that of man is transitively the compendium of all; not, indeed, without a difference, since in each instance, the changing form of the embryo bears the impress of its transitional and complete character, while it ever preserves the promises and prophecy of the being into which it is to be finally evolved.

Every living thing is intimately connected with the evolution of this vast system of animated being. The phenomena of vegetative life ought not, therefore, to be neglected; how much more, when to the vegetative force there is superadded a nervous influence. The lower indications of this nervous influence, as connected with muscular action, or its higher indications as associated with the instinct of inferior creatures, are all deserving of a careful investigation. To be studied philosophically, every organ and function ought to be traced from its rudimentary condition, through all its beautiful adaptations in the organization of the animated tribes which people the earth, the air, and the water, until it arrives at the highest degree of its development in the human organism.

We welcome the appearance of this work, because it is a movement in the right direction. The advantage, or rather absolute necessity of studying the organization of the lower animals in connection with that of man is now universally recognized. A great part, often the best part of the proofs of the most important physiological doctrines, are derived from comparative anatomy.

There are several features in this work, which will insure it a favorable reception. In the text will be found an excellent classification of the invertebrata, from the lowest to the highest forms of organization, accompanied by a bibliography, or list of the best authors who have written on the subject. By this means, the whole work becomes invaluable as a guide-book, and work of reference to the student, who wishes to pursue any "especial line of inquiry and research." To this succeeds a lucid, yet concise exposition of the anatomy and functions of the several organs. The cutaneous, muscular, nervous, circulatory and respiratory systems are described; also the digestive apparatus, the organs of sense, secretion and generation.

The notes give additional confirmation to the statements in the text, and will be found to be rich repositories of valuable information, combining copiousness with brevity.

The work has, however, certain peculiarities, which as they involve an assumption of positions at variance with the generally received views, as to the indefinite character of the limits between the animal and vegetable kingdoms, deserve an especial notice. Thus in Book I. on the "Infusoria and Rhozopoda," page 20, our author says, "cell-structure and free motion are the only two characteristics, in common of the lowest animal and vegetable forms; and since *Schwann* has shown the uniformity of development and structure of animals and plants, it will not appear strange that the lowest conditions of each should resemble each other in their simple cell nature; as to motion, the voluntary movements of Infusoria should be distinguished from those which are involuntary, of simple vegetable forms; a distinction not insisted upon until lately. Thus, in watching carefully the motions of Vorticellina, Trachelina, Kolpodea, Oxytrichina, &c., one quickly perceives their voluntary character. The same is true of the power of contracting and expanding their bodies."

"But in the motions of vegetable forms, other conditions are perceived; and there is no appearance of volition in either change of place or form, their locomotion being accomplished either by means of cilia or other physical causes, not yet well understood. Cilia, therefore, belongs to vegetable, as well as to animal forms; and in this connection it is not a little remarkable, that in animals they should be under the control of volition. However, notwithstanding their free motion from place to place by means of cilia, the vegetable nature of many organisms seemed clearly indicated by the rigid, non-contractile character of their forms. It is from a misapprehension of the true nature of these facts, that some modern naturalists have denied the existence of limits between the two kingdoms."

Without denying "the existence of limits between the two kingdoms," we cannot help saying, that the author has not been as explicit on the subject as he ought to have been. He should have told us what were the facts that induced him to arrive at the conclusion, that the movements of Vorticellina, &c., were voluntary. Such statements should be received with considerable caution, the more so, when they are at variance with the conclusions of some of the best observers of the day. Mr. Owen has never been able to see these voluntary motions. He says, "the motions of the polygastria have appeared to me, long watching them for indications of volition, to be in general of the nature of respiratory acts, rather than attempts to obtain food or avoid danger. Very seldom can they be construed as voluntary, but seem rather to be automatic; governed by the influence of stimuli within or without the body, not felt but reflected upon the contractile fibre; and therefore, are motions which never tire.'' (See Comp. Anat. page 19.)

The fact is, we are not yet in a position to pronounce with the voice of authority, that the animal and vegetable kingdoms are sundered by decisive characteristics.

It was long ago asserted by Bory de St. Vincent and others, that there exists in nature organized bodies, which are animal at one period of their lives, and vegetable at another. This, if true, would forever put an end to the possibility of distinguishing the two kingdoms, when they shall each have arrived at their lowest forms. Its truth has, however, been denied; on the contrary, Kützing, in his recent magnificent work on Algæ, insists that it happens in his Ulothrix Zonata, (Fig.)* He asserts, that in the cells of that plant, there are found minute animalcules, with a red eye-point and a transparent mouth-place; that they are not, in fact, distinguished from Ehrenberg's microglena monadina; these bodies, however, are animals only for a time. At last, they grow into vegetable threads, the lowest joint of which, still exhibits the red-eye point.

This phenomenon, which Kützing assures us he has ascertained beyond all possibility of a doubt, puts an end to the question of whether animals and plants can be distinguished at the limits of their two kingdoms, and sufficiently accounts for the conflicting opinions that naturalists entertain, as to the nature of many of the simpler forms of organization. (Vide Lindley's Vegetable Kingdom.)

Perhaps, the best mark of distinction is that which has been proposed by the chemists, the presence of starch in plants, "an organic compound unknown among the animal creation." By this test, M. Payen has been able to confirm the vegetable nature of certain productions, till lately regarded as Zoophytes, and therefore, as belonging to the animal kingdom.

*Fig. Ulothrix Zonata, after Kützing. 1. A portion of the plant discharging its vegeto-animalcules; 2. The latter much enlarged, and in various states of progress into a thread; 3. A young thread or plant, three or four days old, much less magnified.

In closing this very imperfect notice of the volume before us, we cannot do less than congratulate Dr. Burnett on the very able manner in which he has performed his part as translator and editor. The notes he has added, show his own knowledge on the questions involved to be great; and that he has thought much and profoundly on the subject. The "getting-up" of the book is admirable, and reflects much credit on the publishers.

---

*Transactions of the American Medical Association. Instituted* 1847. Vol. VI.

The fact that our National Association is the representative body of the medical profession of the United States, should cause us to regard with a critical and anxious eye its yearly volume of Transactions. Our scientific character at home and abroad will, in great measure, be estimated by the value of these reports, as it will be naturally inferred, that they would be intrusted only to gentlemen of the highest ability and attainments. It is, therefore, with no ordinary interest that we annually look for their publication. It gives us pleasure to state, that the present volume, though containing no paper of the highest importance, is, on the whole, a creditable one to its authors.

The address of the President, Dr. Wellford, is well and sensibly written. He briefly notices the origin and aims of the Association, and in eloquent language exhibits the happy influences it has already exercised upon the profession. As objects of paramount importance to the advance of medical reform, he earnestly urges the necessity of "a form of organization of both local and State societies," and a proper regulation of the license to practice. Regarding this latter measure, he remarks, "I know of no plan by which this can be effected, other than by a law in each State establishing a Board of Medical Examiners, to whom shall be confided the exclusive power to grant the license to practice within her limits. It should be required to subject each candidate to a rigid examination, and forbidden to grant its certificate to any applicant who was found to be imperfectly qualified. The Board should be unconnected with any school of medicine; but no candidate should be admitted to examination excepting regular graduates of some respectable institution, nor should even then the diploma be recognized, unless it was conferred by a school teaching a full curriculum, and demanding terms of study of sufficient duration to authorize the belief that the diploma emanated from a worthy source, and was bestowed on a worthy recipient." At the close of his address, Dr. Wellford pays a brief and eloquent tribute to the memory of Drake and Horner.

*Report of the Committee on Medical Education.*

The reporter, Dr. Z. Pitcher, reiterates the suggestions of former committees, and observes, "that there is a general desire, not only on the part of the schools, but in the great body of the profession, to come up to the national standard. This disposition has manifested itself in various ways. Some of the Colleges have lengthened their regular terms of lectures, or created new professorships, and added new subjects to the curriculum of studies. Others have established preparatory or supplementary courses of lectures, as a means of meeting the new demand made upon them, in consequence of the agitation of this subject, since the organization of the National Association. The private practitioner has taken new views of duty, and refused to admit into his office the unqualified applicant, and the local societies have voluntarily erected barriers to his taking the initial step in that direction."

These statements, we regret to say, are entirely too much "couleur de rose." We are of opinion that the recommendations of the National Association on the subject of medical education have hitherto effected very little. A few schools, it is true, adopted its views on the requirements for the degree of M. D. The great majority of them, however, paid no attention to them; nor has their so-doing been in any way injurious to their interests. If "the great body of the profession" had really desired a higher standard of medical education, the catalogues of the few complying schools would have testified to the advantage of such a course; whereas, it is well known that these very schools were obliged, in obedience to public opinion and for self-preservation, to make a retrograde movement. We consequently think that Dr. Pitcher is not justified in his statement, "that there is a general desire, not only on the part of the schools, but in the great body of the profession, to come up to the national standard."

Notwithstanding the efforts mode to elevate the character of the alumni of our schools, Dr. P. remarks, that the number of those who fill the ranks of the profession without education is not perceptibly diminished. To remedy this evil, he recommends "the establishment of free colleges for the preparatory and professional education of the young men now scattered over the wild and half cultivated domain of the West." The feasibility of this measure is evinced, he thinks, by the organization of the University of Michigan, the only institution in the country endowed by Government and regulated by State authority. "By the medical faculty of the University, all candidates for the degree of M. D. are rigidly required to comply with the conditions of the National Medical Association, except attendance upon hospital cliniques, and have been notified that, in the future, it is in contemplation to exact the same preparation for admission to the medical as is now prescribed for membership of the department of arts. As an incentive to the prosecution of classical studies, one year will be deducted from the term of medical studies of all students who graduate first in the department of arts."

We have no confidence in the proposed remedy of Dr. Pitcher. It is the general opinion of the profession, that we have already too many Colleges, and that it should be our sole endeavor to extend the requirements of those now in existence. Their further multiplication, on the basis proposed of Government endowment, besides affecting most injuriously the fruits and interests of those which are conducted by private enterprise, would tend, by the spirit of competition they would excite, to lower still further the standard of professional requirements. These objections outweigh, in our opinion, any possible advantages that might be derived from such institutions, and we are constrained, in consequence, to state our disapproval of them.

Dr. Pitcher has a very poor opinion of the value of clinical instruction, as it is now taught in our hospitals, and proposes a new organization to adapt them to the wants of the time. "This could be done by erecting them into schools of practice, with a special faculty, whose plan of instruction should have a direct relation to the cases in their wards, so that each one should become an illustration of the text of the professor."

The Committee conclude their report with the following resolutions:

1. *Resolved,* That the Association reaffirm its formerly expressed opinions on the value and importance of general education to the student and practitioner of medicine, and that it would gladly enlarge its rule on this subject, so as to include the humanities of the schools, and the natural sciences.

2. *Resolved,* That, in the opinion of this Association, a familiar knowledge of the elements of medical science, should precede clinical instruction.

3. *Resolved,* That in order to accomplish the latter, the hospitals, when they shall be elevated to the rank of schools of practice, and the intelligent private preceptor, are the most effectual instrumentalities to be employed.

(To be continued.)

## Figures and Tables

**Figure f1:**